# *In Situ* Breast Cancer and Microchimerism

**DOI:** 10.1038/srep02192

**Published:** 2013-07-12

**Authors:** Jinny K. Eun, Katherine A. Guthrie, Gary Zirpoli, V. K. Gadi

**Affiliations:** 1 Clinical Research Division, Fred Hutchinson Cancer Research Center, Seattle, WA USA; 2 Public Health Sciences Division, Fred Hutchinson Cancer Research Center, Seattle, WA USA; 3 Department of Cancer Prevention and Control, Roswell Park Cancer Institute, Buffalo, NY USA; 4 Department of Medicine, University of Washington, Seattle, WA USA

## Abstract

Microchimeric cells of fetal origin persistent in the maternal circulation post-partum are associated with protection against invasive breast cancer. Here using quantitative genomic methods, we evaluated for the presence of male fetal microchimerism in buffy coat cells from women with a prior history of breast carcinomas in situ (CIS) and in healthy controls. Fetal microchimerism was detected in 75 of 88 controls (85%) and in 57 of 89 CIS patients (64%). The odds ratio for protection against non-invasive breast disease was 0.26 (95% confidence interval 0.12–0.56; p < 0.001 adjusted for age and body mass index). Similar to women with invasive breast cancer, women with CIS who are naturally at high risk for future invasive disease were deficient for fetal microchimerism. In addition to autologous anti-tumor immune responses, the maintenance of haploidentical microchimerism may impart an allogeneic edge in immunosurveillance.

Breast carcinomas in situ (CIS) are non-obligate precursors for invasive disease. Though no true prospective natural history studies of CIS are at this time ethically feasible, a woman diagnosed with non-invasive breast carcinoma optimally managed with breast conserving surgery only is estimated to retain an 8–10 year risk of recurrent disease ranging from 26–31% in her remaining breast tissue^1^. Understudied compared to invasive breast cancers, the etiologic factors lending to development of in situ cancers can inform greatly about more aggressive forms of disease. Similar to invasive breast cancer, available evidence supports a protection against developing in situ breast cancer when women are parous^2^. In addition to autologous immune responses against neoantigens and direct hormonal changes to breast tissues originating during pregnancy that are known to afford an advantage against cancer^3^, we have been evaluating a new dimension to this protection, fetal microchimerism. Fetal microchimerism describes the small numbers of haploidentical cells that transit during pregnancy and persist in a woman's circulation and tissues long-term. In prior studies published by our group, fetal microchimerism was both associated with freedom from breast cancer when present in the circulatory system^4^,^5^ and in breast tissue^6^. Moreover, when women are deficient in fetal microchimerism, they are at a higher risk for developing a future breast cancer^7^. Because pre-malignant or pre-invasive disease can be present years prior to developing an invasive cancer, we sought to determine if women with pure in situ breast cancers were deficient for fetal microchimerism. Specifically, if our hypothesis is correct, it suggests that there is likely a fundamental failure of acquiring or maintaining chimeric cells from the fetus in women with breast disease or cancer as opposed to a loss of it during progression towards overt disease.

## Results

Peripheral blood cell buffy coat DNA from 100 women with a history of CIS and 100 healthy control women (also referred to as probands) were obtained from the Roswell Park Cancer Center Data Bank and BioRepository^8^. Probands included in our study were recruited to the biorepository over a 6-year span from 2004–2010 and donated their blood specimens a median of 34 days after diagnosis. Controls were matched to case probands on the basis of gender, age (in 5-year blocks), parity (yes vs. no), and race. Quantitative PCR was performed over a 7 month span from June 2011 to Jan 2012. Nine case and 12 control specimens were excluded from analysis because DNA quality (n = 9) or quantity (n = 12) was insufficient for PCR. We utilized a real-time quantitative PCR assay to detect a y-chromosome sequence of *DYS14* to identify male DNA in probands' buffy coat DNA. Following case status-blinded analysis of quantitative PCR results, data from 91 CIS and 88 control subjects were available for analysis. Two CIS probands were excluded from final analysis because male DNA quantities in these two women amplified substantially beyond the highest point on the calibration curve (500 *DYS14* containing genome equivalents). Though precise estimates could not be ascertained, these patients' peripheral blood cells were composed of 27% and 80% male cells. We speculate hematopoietic macrochimerism originating for these two women while they were themselves in utero from a vanished twin. The remaining 89 CIS probands were included in the final analysis. The two cohorts were similar with respect to all factors shown in Table 1. The total number of cell equivalents tested for detection of male microchimerism was higher in the CIS cases than in the controls, although this difference was not statistically significant (mean 9.1 × 10^4^ (95% confidence interval (CI), 8.5–9.6 × 10^4^) in cases and 8.4 × 10^4^ (95% CI, 7.9–8.9 × 10^4^) in controls; p = 0.05).

Overall, male microchimerism was detected in 132 women, including 75 of 88 controls (85%) and 57 of 89 CIS patients (64%). Table 2 shows the prevalence of male microchimerism and the association of male microchimerism and risk of CIS. Compared to women who were microchimerism-negative, women harboring microchimerism were less likely to have had CIS (OR 0.26, 95% CI 0.12–0.56; p < 0.001) in a model adjusted for age and BMI. In subset analysis by number of children, the association of microchimerism and risk of CIS was stronger in women with two or more children than in nulliparous or uniparous women (Table 2). A test for interaction across subgroups according to number of children (0, 1, 2, 3 or more children) showed a decreasing trend in the odds ratios (p = 0.07). Table 3 shows subset analysis by age at first birth among women with at least one child, where the association of microchimerism and risk of CIS was significantly stronger in women who first gave birth before age 30 years compared to those over 30 (p = 0.04 for interaction).

**Figure 1:**
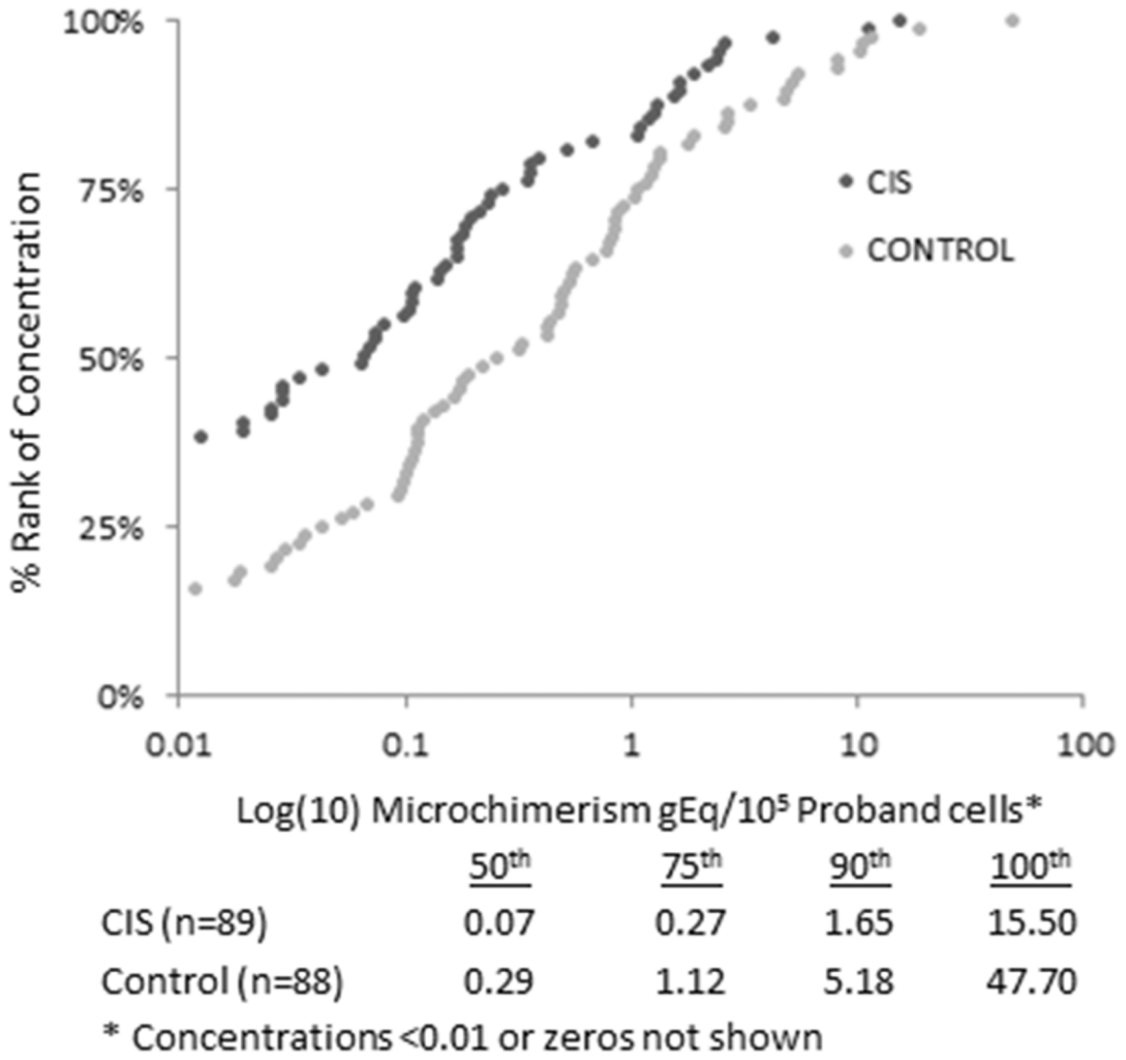
Microchimerism concentrations by CIS/Control status. Quantities (fetal genome equivalents (gEq)/10^5^ proband cells) on X-axis are base-10 log transformed with zeros (n = 45) and small concentrations (n = 2) excluded to better demonstrate differences at various percentile ranks.

Median concentrations were 0.29 versus 0.07 male chimeric cells per 10^5^ host cells in control and case subjects, respectively (Figure 1) and differed substantially at all percentile ranks. Logistic regression models used to model the primary outcome of CIS with presence of fetal microchimerism may substantially underestimate the impact of any association because it does not account for quantitative differences between groups. We therefore applied Poisson modeling (because of the rightward skew of the data) to better capture quantitative differences between groups. Using this approach, the rate of microchimerism detection was significantly lower in women with history of CIS than in the healthy women (p < 0.001) in a model adjusted for age, education level and moderate exercise (Table 4). Moreover, the association of microchimerism and risk of CIS more evidently varied by number of children compared to ORs shown in Table 1; a test for interaction showed that there was a statistically significant *increasing* trend in the rate ratios (p = 0.02).

The prevalence of microchimerism and its association with characteristics of the CIS cases is shown in Table 5. There was no indication of variation in microchimerism prevalence according to these characteristics. Progesterone receptor results are not shown because they were identical to those for estrogen receptor.

## Discussion

We report that women with in situ breast cancer are deficient in carriage of male microchimerism of presumed pregnancy origin at rates comparable to those observed in women with invasive breast cancer. Combined with our prior prospective study of microchimerism in healthy women who later develop invasive breast cancer^7^, the current study also serves as evidence that the absence of microchimerism is not a result of having developed breast cancer but more likely a predisposing condition towards it. The data collectively indicate a primary failure of microchimerism acquisition during pregnancy or alternatively a loss well prior to developing non-invasive or invasive disease. The ideal study design to verify the temporality of the microchimerism loss with respect to cancer development would be a longitudinal serial study beginning with pregnancy completion; unfortunately, to the best of our knowledge a mature resource of this kind does not yet exist. In considering fetal microchimerism as a biomarker, this report is now the 6^th^ study available demonstrating this consistent observation, all of roughly similar magnitude^4^,^5^,^6^,^7^,^9^.

Though contamination of specimens with male DNA cannot be absolutely excluded, strengths of the study are that laboratory hygiene practices, a single female operator, and blinding insure non-differential uncontrolled effects among the groups and further bolsters confidence in the findings. Moreover, the presence of potential contamination would bias against our study because it would likely dilute differences between groups. It is noteworthy that the rate of microchimerism detection in controls was higher than observed in prior invasive cancer association studies. We attribute the higher detection rate in our prospective study^9^ and the current report to the use of more modern PCR rigs with known higher sensitivity and improved performance characteristics. Another consideration is that gravidity was not recorded for participants in the registry. Thus, we are not sure of the impact, if any, of miscarriages and abortions on the microchimerism prevalence in the cohort. The present study was powered to primarily identify an association of microchimerism with CIS versus matched controls based on assumptions derived from prior studies already published for invasive disease. However, there was insufficient power to draw firm conclusions regarding CIS specific characteristics such as laterality or tumor subtype, but generally no striking differences emerged in these analyses. Unfortunately, to the authors' knowledge no other bio-repositories with appropriate specimens for microchimerism testing in CIS are available other than the Roswell Park resource used here, which we nearly exhausted for unique case specimens.

The mechanism by which microchimerism might protect against breast malignancy remains undefined but a leading hypothesis is that such cells are involved in allogeneic immune surveillance. In a sense, haploidentical fetal cells crossing over during pregnancy might serve as a natural version of microtransplant. As support that microchimeric populations of cells can be immunologically active participants in graft-versus-tumor effects absent of any concomitant graft-versus-host disease, Guo, et al recently reported that haploidentical related donor hematopoietic cell microtransplantation without an adequate conditioning regimen or immunoprophylaxis was an effective form of consolidative therapy for acute myelogenous leukemia with clear demonstration of graft-versus-tumor and host-versus graft effects^10^. Moving forward and with additional studies, we pose the question whether primed or unaltered haploidentical cell infusions, possibly using child-origin cells could be considered as form of immunotherapy for women at high risk of breast cancer recurrence following otherwise definitive therapy for *in situ* or invasive disease.

## Methods

### Ethical considerations

Research subjects originally signed consent forms approved by the Roswell Park Institutional Review Board at the time of enrolment into the Data Bank and BioRepository^8^. Because only non-identifiable specimens were provided to investigators, the current research is considered non-human subjects research.

### Fetal microchimerism testing

Each genomic DNA specimen was estimated for total DNA content and purity by spectrophotometry. Presence and quantity of fetal microchimerism was determined by targeting the y-chromosome sequence *DYS14* by PCR^11^. Briefly and with attention to modifications of the original technique, aliquots of genomic DNA (2–3.5 × 10^4^), were tested using TaqMan chemistry performed on an ABI PRISM 7900 PCR rig for beta-globin (2 aliquots) to determine total proband genomes and for *DYS14* (6 aliquots) to determine total microchimeric genomes present. Amplifications for total and microchimeric genomic DNA were plotted against calibration curves for both the beta-globin and *DYS14* assays to determine quantities and final results were expressed as a ratio of microchimeric cells per 1.0 × 10^5^ maternal genomic equivalents. All genomics workflow was performed by a single female operator (JKE) blinded to case-control status of specimens to prevent laboratory contamination of male DNA sequences and bias, respectively.

### Statistical analysis

Differences in subject characteristics between CIS cases and controls were assessed via t-test for continuous variables and Chi-squared test for categorical factors. Pre-study power calculations for the primary analysis of presence of microchimerism informed that 100 case and control specimens each provided 83% power to detect a 0.2 prevalence difference from the assumed-true rate of 0.5 in controls, based on a chi-squared test with 2-sided type I error level of 5%. Logistic regression models were used to estimate the association between the primary outcome of disease status and the presence of fetal microchimerism. Subset analyses were performed according to number of children and age at first birth; exact logistic regression was used for analysis in small samples.

Fetal microchimerism concentrations were also analyzed by disease status. By definition, microchimeric cells occur at low concentrations; therefore the data distribution is skewed to the right and approximates a Poisson distribution. For this reason, we analyzed the concentrations as the outcome in log-linear regression models, estimating a rate of microchimerism detection as the number of genome equivalents of fetal DNA as a proportion of the number of maternal cells tested. Negative-binomial models were fit to account for the higher level of variability in the data than expected in a Poisson model; interpretation of the resulting estimates is identical to those of a Poisson model.

Factors examined as potential confounders or effect modifiers included age, body mass index (BMI), education, moderate exercise (none, low, and high, defined as at least 30 minutes per session on 3 or more days/week), 1st degree family history of breast cancer, age at menarche, age at first birth, number of births, history of breastfeeding, menopausal status, and menopausal female hormone use. Covariates were selected a priori based on having an established or suspected causal association with breast cancer incidence. We also considered the number of cell equivalents tested for detection of fetal microchimerism as a potential confounder. A factor was defined as a confounder if there was a discrepancy of 10% or more in the estimated coefficient of interest between the multivariable model including the factor and the model without it.

Additional analyses were conducted to examine whether CIS-specific features were associated with fetal microchimerism prevalence among the cases. Presence of fetal microchimerism was treated as a binary outcome in logistic regression models, with various disease characteristics as the predictors.

P-values from regression models were derived from the Wald test or an exact binomial test for small sample sizes in prevalence analysis. No adjustments were made for multiple comparisons. Analyses were performed on SAS software version 9 (SAS Institute, Inc., Cary, NC).

## Author Contributions

J.K.E., K.A.G., G.Z. and V.K.G. participated in the design of the experiments, analysis of results, and writing of the manuscript. J.K.E. performed all bench experiments.

## Figures and Tables

**Table 1:** Subject characteristics by disease status

	Cases (N = 89)	Controls (N = 88)
	N (%)	N (%)
Age (years)		
29–44	16 (18)	16 (18)
45–54	34 (38)	28 (32)
55–64	26 (29)	30 (34)
≥65	13 (15)	14 (16)
Education		
Below or at high school	25 (28)	18 (20)
Some college	31 (35)	34 (39)
College graduate	14 (16)	18 (20)
Advanced degree	19 (21)	18 (20)
Body mass index (kg/m^2^)		
<25	35 (40)	26 (30)
25–29	33 (38)	31 (36)
≥30	19 (22)	30 (34)
Unknown	2	1
Smoking status		
Never	48 (55)	47 (53)
Current	14 (16)	10 (11)
Former	25 (29)	31 (35)
Unknown	2	0
Moderate exercise		
No	52 (60)	49 (56)
Low	16 (18)	21 (24)
High	19 (22)	18 (20)
Unknown	2	0
Family history of BRCA		
No	71 (80)	70 (80)
Yes	18 (20)	18 (20)
Age at menarche (years)		
≤11	13 (15)	16 (18)
12	27 (32)	25 (29)
13	27 (32)	28 (32)
≥14	18 (21)	18 (21)
Unknown	4	1
Number of births		
0	23 (26)	23 (26)
1	11 (12)	11 (12)
2	22 (25)	20 (23)
3	18 (20)	19 (22)
4 or more	15 (17)	15 (17)
Age at first birth (years)		
19 or younger	9 (14)	12 (19)
20–24	19 (29)	27 (42)
25–29	25 (38)	15 (23)
30 or older	13 (20)	10 (16)
Unknown	0	1
No children	23	23
Number of children breastfed		
No children	23 (26)	23 (26)
0	29 (33)	21 (24)
1	14 (16)	14 (16)
2	12 (13)	15 (17)
3 or more	11 (12)	14 (16)
Unknown	0	1
Menopausal status		
Premenopausal	43 (48)	37 (42)
Postmenopausal	46 (52)	51 (58)
Menopausal hormone use		
No	66 (74)	64 (73)
Yes	23 (26)	23 (26)
Unknown	0	1

**Table 2:** Odds ratios (OR) of CIS by microchimerism prevalence, for all subjects and by number of children

		Proportions by			
		Disease Status (%)			
Parity	Presence of microchimerism	Cases	Controls	OR (95% CI)	p-value
All subjects	No	32 (36)	13 (15)	1.0	
	Yes	57 (64)	75 (85)	0.26 (0.12–0.56)	<0.001*
No children	No	6 (26)	5 (22)	1.0	
	Yes	17 (74)	18 (78)	0.79 (0.20–3.06)	
1 child	No	3 (27)	3 (27)		
	Yes	8 (73)	8 (73)	1.00 (0.10–9.94)	
2 children	No	10 (45)	1 (5)		
	Yes	12 (55)	19 (95)	0.06 (0.01–0.58)	
≥3 children	No	13 (39)	4 (12)		
	Yes	20 (61)	30 (88)	0.21 (0.04–0.81)	0.07*

*for all subjects, adjusted for age and BMI; in subset analysis, unadjusted test for trend.

**Table 3:** Unadjusted OR of CIS by microchimerism prevalence, according to age at first birth in parous women

		Proportions by Disease Status (%)		
Age at 1^st^ birth	Presence of microchimerism	Cases	Controls	OR (95% CI)
<30 years	No	21 (40)	4 (7)	1.0
	Yes	32 (60)	50 (93)	0.12 (0.04–0.39)
≥30 years	No	5 (38)	4 (40)	1.0
	Yes	8 (62)	6 (60)	1.07 (0.20–5.77)

**Table 4:** Rate ratios (RR) of CIS by microchimerism detection, for all subjects and by number of children

Parity	Rate Ratio (95% CI)	p-value
All subjects	0.47 (0.31–0.71)	<0.001*
No children	0.28 (0.12–0.64)	
1 child	0.47 (0.20–1.11)	
2 children	0.22 (0.10–0.49)	
≥3 children	0.97 (0.53–1.76)	0.02

*for all subjects, adjusted for age, education level (college or more vs. less than college) and moderate exercise; in subset analysis, unadjusted test for trend.

**Table 5:** Among CIS cases, unadjusted OR of microchimerism prevalence according to disease characteristics

	No microchimerism (N = 32)	Microchimerism (N = 57)	
	N (%)	N (%)	OR (95% CI)
Laterality			
Left origin of primary	19 (59)	31 (54)	1.0
Right origin of primary	13 (41)	26 (46)	1.23 (0.51–2.95)
Estrogen receptor			
Positive	12 (75)	26 (72)	1.0
Negative	4 (25)	10 (28)	0.87 (0.23–3.33)
Test not done	14	20	–*
Unknown	2	1	–*
Grade			
Well differentiated	2 (18)	3 (27)	1.0
Moderately differentiated	3 (27)	4 (36)	0.89 (0.09–9.16)
Poorly differentiated	6 (55)	4 (36)	0.44 (0.05–3.98)
Unknown	21	46	–*
Histologic Subtype			
Ductal Carcinoma In Situ	22 (69)	43 (75)	1.0
Lobular Carcinoma In Situ	3 (9)	6 (10)	0.97 (0.17–5.04)
Other^	7 (22)	8 (14)	1.71 (0.48–6.12)

*Patients with unknown values are excluded from analysis.^includes in situ subtypes of comedo, papillary, intracystic, and cribiform.
